# The Mediterranean and Nordic Diet: A Review of Differences and Similarities of Two Sustainable, Health-Promoting Dietary Patterns

**DOI:** 10.3389/fnut.2021.683678

**Published:** 2021-06-25

**Authors:** Željko Krznarić, Irena Karas, Dina Ljubas Kelečić, Darija Vranešić Bender

**Affiliations:** ^1^University Hospital Zagreb, Department of Internal Medicine, Clinical Unit of Clinical Nutrition, Zagreb, Croatia; ^2^Zagreb School of Medicine, Zagreb, Croatia

**Keywords:** Mediterranean diet, Nordic diet, olive oil, rapeseed oil, plant-based, chronic disease prevention

## Abstract

The Mediterranean diet (MD) and the Nordic diet (ND) share more similarities than differences. Both diets are based on typical local and seasonal foods, share similar nutritional recommendations based on plant-based dietary principles, and are both now orienting toward environmental protection and sustainability. The main difference between the two diets is the primary fat source. Olive oil is the synonym for MD while the ND uses more rapeseed/canola oil. While longitudinal epidemiological studies support adherence to MD as a way to prevent chronic diseases, ND still needs more such studies because the current results are discrepant. Notably, studies that assessed the association between both diets and lower risks of chronic diseases, disability, and mortality from specific and all causes, implied that ND could also have an advantageous effect as MD. Hopefully, there will be more longitudinal and large prospective studies in the future that will provide more evidence-based recommendations.

## Introduction

In countries around the world, balanced diets that are based on practical, sustainable, and health-promoting dietary guidelines, which support locally available food, represent typical preventive policies (1, 2). Since the Food and Agriculture Organization (FAO) predicts that food production will increase by at least 60% by 2050, radical changes in food production and consumption are needed to conserve natural resources for future generations while providing sufficient food for a growing global population (3).

Both the Mediterranean diet (MD) and the Nordic diet (ND) have similar dietary recommendations, are considered to be plant-based, and are oriented toward environmental protection and sustainability. The objective of this mini-review is to show that there are more similarities to the two diets than differences. Although the north and the south of Europe differ significantly in their climate, which undoubtedly affects the types of food that can be grown, both diets are so similar in their general guidelines that the main notable difference between the two is in the oil that is used in everyday consumption. Since longitudinal and large prospective studies for ND are still lacking, it is still not so easy to provide evidence-based recommendations just for the ND. We reviewed the studies that compared the effects of both diets on human health, which showed that greater adherence to ND is associated with lower all-cause and cardiovascular mortality and lower incidence of disability. These studies indicate that ND could have as beneficial effects as the MD. With this in mind, we believe that both diets can be implemented and perhaps even alternated as a part of a healthy lifestyle.

## Composition and Concept of the ND and MD

The Nordic Diet is based on traditional ways of eating in the Nordic countries: Denmark, Norway, Sweden, Finland, Greenland, and Iceland. Certain foods and preparation methods are relatively the same in mentioned countries and include berries (e.g., lingonberry), cabbage, apples, pears, root vegetables, oats, rye, and fermented milk. All of these traditional Nordic foods have been associated with beneficial health effects (4, 5). As meat is the least environmentally friendly food, ND recommends an increased intake of legumes which helps increase the amount of protein in the diet but also has a beneficial effect on reducing the pressure on the environment (6). Moreover, Nordic countries have a rich marine archipelago, which is a great source of fish and other seafood that represent an important part of the diet (1, 6). The most common source of added fats in the ND is rapeseed (also known as canola oil) which is produced from the Rapeseed plant (*Brassica napus*), a member of the Cruciferae family. This can probably be ascribed to the geographical location of the countries since the rapeseed plant is predominantly cultivated in its winter form. The collaboration of Nordic countries for several decades has resulted in setting guidelines for dietary composition and reference values for the nutrient intake through the conjoint publication of the Nordic Nutrition Recommendations (NNR). This is one of the most comprehensive regional collaborations of the world, involving Denmark, Finland, Iceland, Norway, Sweden, the Faroe Islands, Greenland, and Åland (7, 8). According to the NNRs, the recommended daily energy distribution (as energy percent, E %) for macronutrients is: 25–40% of total energy intake should be derived from fats, 45–60% from carbohydrates, and 10–20% from protein (7). Since the ND includes several countries, the food-based dietary guidelines (FBDGs) slightly vary for every Nordic country. The daily quantitative recommendations for fruits, vegetables, starchy foods (wholegrain cereals or wholemeal alternatives), fish, and red meat are somewhat similar, while milk and dairy products are not specified in quantitative recommendations for all countries, but all are in concordance that low-fat dairy products should be the preferred choice. All countries advise using “softer and healthier fat” like plant oils and soft margarine, and limitation of salt to 5–6 g/day. An average daily intake of 1.5 L of water or other unsweetened liquids is recommended (9).

The Mediterranean diet incorporates traditional living habits of people from countries surrounding the Mediterranean Sea; it varies by country and region and has a range of definitions, and is the result of the long tradition of a century of sharing goods, food, and culture between countries in the Mediterranean area. Due to contemporary lifestyle and environmental challenges, the traditional MD pattern had to be updated, especially on the topics of local, seasonal, and minimally processed food consumption, sustainability, and cost of food, while adapting to socio-economic, cultural, and geographical contexts. According to MD, everyday main meals and snacks should contain cereals, vegetables, and fruit, while low-fat dairy products should be consumed in moderation. Two or more servings of fish and white meat, with weekly consumption of various plant proteins, is recommended. Red and processed meat should be reduced in quantity and frequency of consumption. A daily average of 1.5–2 L of water or other unsweetened, low-sodium liquids should be ensured. The main source of dietary fat is olive oil, especially extra virgin olive oil (EVOO) (10). Studies vary when trying to define daily energy distribution for macronutrients; according to Davis et al. (11), the MD provides close to 37% energy from total fat, 15% from protein, and at least 43% energy from carbohydrates (11).

The MD and the ND are considered as “plant-based” eating patterns, both recommend choosing more of the proteins from plant sources by recommending a higher intake of fruits, vegetables, grains (especially whole grains), legumes, nuts, and seeds, while limiting consumption of red and processed meat (12). The notable point of difference is the oil used in each diet, while MD is based on olive oil (preferably EVOO), the ND mainly uses rapeseed (canola) oil (RO) (13). The main bioactive components of EVOO, which affect sensory and contribute to its nutritional characteristics are MUFA, PUFA, phytosterols, polyphenols, pigments, tocopherols, squalene, triterpenic acids, and dialcohols. Olive oil has a high content of MUFA, especially oleic acid (18:1 ω-9), whose health-promoting effects came from cardiovascular prevention trials that showed versatile biological effects: modifications of plasma lipids and lipoprotein patterns, impact on membrane composition and fluidity of blood cells, inhibition of coagulation, improvement of glucose homeostasis and blood pressure, and reduction of inflammation and oxidative states in fasting conditions. Olive oil is high in squalene, which besides anticancer and antioxidant properties, also has an essential role in cholesterol metabolism in humans. Over 100 different types of biophenols have been reported in olive products, from which hydroxytyrosol (HT), its metabolites, and oleocanthal (known for its anti-inflammatory and chemotherapeutic properties, neuroprotective and anti-rheumatic effects) have been extensively researched (14–16). Phenols are known for their antioxidant, anti-inflammatory, and antimicrobial activities, and epidemiological and observational studies have shown their effectiveness in the prevention of inflammatory and chronic human conditions such as cardiovascular disease (CVD), cerebral diseases, and cancer (14). RO has a very good lipid profile, it is characterized by a low level of SFA and significant amounts of MUFA and PUFA, and has a notably higher level of α-linolenic acid (ALA) than olive oil, which is connected to cardioprotective benefits (15, 17, 18). RO contains fewer phenolic compounds, but the amount of phytosterols and tocopherols is higher (16, 17). RO also contains pigments (chlorophylls) and other trace elements i.e., ubiquinone (Coenzyme Q10), a compound involved in energy production and prevention of peroxidative damage to membrane phospholipids and free radical-induced oxidation (19, 20). Significant differences between bioactive compounds in EVOO and RO, apart from the basic source material, are the result of different production methods: EVOO is produced by mechanical extraction, while RO production needs more processing (solvent extraction, degumming, neutralization, bleaching, and deodorization). Side effects of this production are depletion of certain phytosterols, tocopherols, and other bioactive compounds that were present in the rapeseeds at the beginning (17, 18).

## Discussion

The Mediterranean diet has been extensively assessed in relation to chronic diseases. Because of the recognition that clinical and longitudinal epidemiological studies are needed for evidence-based recommendations, the publications on this subject have increased significantly since 1985. Some of the intervention studies such as the Medi-RIVAGE study in France, the PREDIMED (*Prevencion con DietaMediterranea*) study in Spain, and prospective cohorts such as the European Prospective Investigation into Cancer and Nutrition (EPIC) study and the SUN study have been analyzing the value of MD (20). On the other hand, the effects of the ND on major chronic diseases that were examined through three large prospective cohorts in Denmark, Sweden, and Finland resulted in inconsistent outcome. Still, studies that were based on the comparison of both diets and their potentially beneficial effect on chronic diseases, disability, specific-cause, and all-cause mortality, implied that ND also has a beneficial effect as MD.

The PREDIMED study, a multicenter, randomized prevention trial assessed effects of the low-fat diet, MD rich in olive oil, and MD rich in tree nuts on CVD. The results showed that MD (either with virgin olive oil or a mixture of nuts), compared to a low-fat diet, resulted in lower blood pressure, decreased insulin resistance, reduced concentrations of inflammatory molecules, and improved lipid profiles, after 3 months of follow-up (20). However, even though this is one the most influential randomized trials, in June 2018 the trial was retracted and republished because serious protocol deviations were detected; the original was substituted with a reanalysis that presented PREDIMED as a non-randomized study and in the end gave similar estimates for the primary endpoint (21). The EPIC study showed that greater adherence to MD was associated with a small reduction in the risk of developing type 2 diabetes (T2D) (22), while the EPIC study in Greece on the elderly European population and survival showed that greater adherence to the MD was connected to lower mortality, an inverse association was observed for overall mortality, coronary heart disease, and cancer mortality (20). The UK-based EPIC-Norfolk prospective cohort which examined the relationship between MD and incidence of CVD showed that greater compliance with MD was associated with lower incidence of CVD and mortality (23). The SUN study on MD in the primary prevention of nutrition-related chronic disease that included more than 22,000 participants in Spain from 1999 to February 2018, validated self-reported data on lifestyle, diet, and clinical diagnosis. The study outcomes showed that high adherence to MD is associated with reduced incidence of mortality, CVD, T2D, weight gain, metabolic syndrome, depression, cognitive decline, nephrolithiasis, and data even suggested that it may also enhance fertility (20, 24). The Medi-RIVAGE intervention study compared the effects of MD to a low-fat diet in subjects at high cardiovascular risk, which showed that participants on MD had better improvements in the majority of the cardiovascular risk factors compared to those on a low-fat diet after 3 months of follow-up (20). It also improved postprandial lipaemia in both genders at moderate cardiovascular risk (25, 26).

The Diet, Cancer and Health cohort study in Denmark showed that ND was associated with a lower risk of T2D for both men and women (27), and women who strongly complied with this diet had a lower incidence of colorectal cancer (CRC), but no significant effect was found for men (28). From the same cohort study, men and women with cases of stroke were identified from the Danish National Patient Register and better compliance to the ND was related to a lower risk of stroke, which leads to the conclusion that the ND could be suggested for stroke prevention (29). The Swedish Women's Lifestyle and Health cohort examined the association between ND incidence of overall CVD, CRC, and risk of breast cancer, and found no association between adherence to the ND and the risk of aforementioned diseases (30–32). However, prospective analysis in this cohort that examined the association of ND and total and cause-specific mortality, found that higher adherence to ND was associated with a 6% lower total mortality per 1 point increment in the adjusted models (32). Additionally, a prospective study that included two independent Finnish studies (the Helsinki Birth Cohort Study and the Health 2000 Survey) found no statistically significant association between adherence to the ND and incidence of T2D during 10 years of follow-up and suggested a larger prospective study to get stronger estimates (33, 34).

The EPIC-Potsdam study in Germany (part of the European-wide multicenter EPIC study) was designed to explore the relationship between both diets and the risk of chronic diseases (T2D, myocardial infarction (MI), stroke, and cancer). With a mean of 10.6 years of follow-up, the MD displayed inverse associations with the risk of developing T2D and MI (the latter only among women), while the ND demonstrated an inverse (but not-statistically profound) association with MI risk and stroke (the latter only among men). No relationship was observed for both diets with the incidence of cancer (35). A prospective cohort study in Northern Germany investigated the connection between MD and ND with all-cause mortality in long-term CRC survivors where the key findings showed a statistically significant association of MD with better overall survival, and the same can be implied for ND in the multivariable-adjusted model (36). A longitudinal study on subjects who belonged to the Helsinki Birth Cohort Study that analyzed the relationship between the two diets with the incidence of disability in a 10-year follow-up showed that individuals with greater adherence to the ND had lower mobility limitations and difficulties to perform self-care activities, while those with greater adherence to the MD had lower disability incidence, but with no statistically significant association. The study implies that the ND could decrease the probability of disability incidence and that the MD could also be helpful in the matter, but more longitudinal and intervention studies are needed (37). In a study from the Swedish Mammography Cohort, two diet scores [modified Mediterranean diet score (mMED) and Healthy Nordic Food Index (HNFI)] and their conjoined effect related to all-cause and cause-specific mortality (cancer, CVD, and ischemic heart disease) were assessed. It should be noted that the mMED was adapted to fit the non-Mediterranean settings, and the used HNFI was created in Denmark (consequently, this HNFI may lack certain ND features distinctive for Sweden). This study demonstrated that mMED was strongly associated with lower cause-specific mortality than HNFI, but both were inversely associated with lower total all-cause and cardiovascular mortality. This result may seem to be contrary to the results of The Swedish Women's Lifestyle and Health cohort where the adherence to the ND was not related to the risk of CVD, but presented a 6% lower total mortality per 1 point increment which was limited to cancer and non-CVD causes. Different results may be due to a greater number of CVD cases in this cohort and older subjects with greater BMI. The results of this cohort may indicate a greater advantage to be adherent more to the mMED than HNFI, but it should be taken into account that in this cohort, the HNFI was perhaps not the true embodiment of the ND and may still need improvement (38). The summation of the results of studies comparing the effect of both diets on human health is shown in Table 1.

**Table 1:** Studies that compare the effects of both diets on human health.

**Name of the study**	**Objective**	**Method**	**Results**
The EPIC-Potsdam study (a prospective cohort based in Germany as part of the European-wide multicenter EPIC study)	The aim was to investigate the association between the ND and the MD with the risk of chronic diseases (type 2 diabetes (T2D), myocardial infarction (MI)	27,548 participants were recruited between 1994 and 1998. After exclusion of prevalent cases, baseline adherence to the diet score was evaluated, with application of Cox regression models to examine the association between the diet scores and the incidence of major chronic diseases	The ND showed a statistically non-significant inverse association with incidence of MI in the overall population and of stroke in men. Adherence to the MD was associated with lower incidence of T2D. In women, the MD score was also inversely associated with MI. No association was observed for any of the scores with cancer
PopGen CRC survivor cohort (prospective cohort study in Northern Germany)	Aim was to investigate the association of the two diets with all-cause mortality in long-term CRC survivors	Diet was assessed at a median time of 6 y after cancer diagnosis in 1,404 CRC survivors (median age: 69 y; 56% men) by using a semiquantitative food-frequency questionnaire. Cox proportional hazard models were used to assess associations of the diets with all-cause mortality	In multivariable-adjusted models, higher adherence to the MD was significantly associated with lower all-cause mortality. Similarly, the ND was inversely associated with all-cause mortality (when the highest was compared with the lowest index quartile) and when modeled as a continuous trait
The Helsinki Birth Cohort Study	Aim was to assess the association of the ND and the MD with incident disability with a follow-up of 10 years	962 home-dwelling men and women, mean age 61.6 years, who were free of disability at baseline, participated in a clinical examination	The likelihood of having mobility limitations and difficulties in self-care activities were lower among those who had better adherence to the ND. Greater adherence to MD was associated with a lower disability incidence (however, the association was not statistically significant)
The Swedish Mammography Cohort	The combined effect of the two diet scores and association with all-cause and cause-specific mortality (cancer, CVD and ischemic heart disease)	The study included 38,428 women (median age of 61 years) where diet and covariate data were collected in a questionnaire	Higher adherence to MD was associated with lower mortality and compared with ND, was more strongly associated with a lower cause-specific mortality. Both MD and ND were inversely associated with all-cause and cardiovascular mortality. Cancer mortality was not independently associated with ND, whereas higher adherence to the MD was associated with lower cancer mortality

Much was discussed about the health benefits of MD and ND, but both also illustrate how a diet can also have a positive impact on the environment and the nitrogen cycle (39). It is well-known that current food systems have a significant effect on the environment; food production, processing, preservation, and distribution of waste, all consume a significant amount of energy and resources, which contributes to about 32% of total greenhouse gas emissions (GHG) from human activities. A recent study based on MD and ND proposed a new way to assess the environmental impact of the diets, by introducing an environmental hourglass (EH) approach. The idea is to facilitate translating healthy dietary recommendations (that also take the regional context and cultural diversity into account) into practical dietary advice that is both sustainable and environmentally friendly. The EH approach describes the production of GHG through weekly consumption of the recommended dietary intake. The visual concept of the EH depicts the foods that should be consumed frequently every day at the bottom, while the foods that are supposed to be consumed one to three times per week are at the top of the hourglass. Therefore, fruits and vegetables were placed at the bottom, after which came cereals and potatoes, followed by milk and dairy products, and finally at the top are meat, fish, eggs, and legumes. Both MD and ND had similar GHG impact, even though the distribution of food and the contribution of individual food categories differed: vegetables contributed most to the overall impact in MD, and for ND, were the wholegrain/cereals; also, the recommended weekly amount of protein-rich foods, as well as the distribution of specific food items in this group, varied in both diets. The study demonstrated that appropriate food choices in accordance with the MD and ND may reduce some of the adverse effects of food production on the environment (40).

## Conclusion

Mediterranean diet is not only perceived as a healthy diet but also as a way of life (with country-specific variations). MD and ND share similar nutritional recommendations and prefer seasonal, locally available foods while taking into account cultural heredity, sustainability, and preservation of the environment. We believe that both of these diets can be implemented and perhaps even alternated as a part of a healthy lifestyle, regardless of the geographical location. MD and its effects on health have been vastly studied, but similar longitudinal epidemiological studies for the ND are still lacking. Although some focus only on specific bioactive compounds in the diet, it should probably be better to assess ND as a whole; because the overall impact on health comes not only from one isolated bioactive component but rather a combination of different compounds from food that we eat every day and the whole lifestyle.

## Author Contributions

ŽK and DV designed the concept of the mini-review. IK and DL interpreted the data. IK and DV drafted the manuscript. All authors performed the critical analysis of the manuscript, approved the final manuscript, read, and agreed to the published version of the manuscript.

## Conflict of Interest

The authors declare that the research was conducted in the absence of any commercial or financial relationships that could be construed as a potential conflict of interest.

## Abbreviations

MD: Mediterranean diet
ND: Nordic diet
NNR: Nordic Nutrition Recommendations
EVOO: extra virgin olive oil
RO: rapeseed oil
MUFA: monounsaturated fatty acids
PUFA: polyunsaturated fatty acids
ALA: α-linolenic acid
CVD: cardiovascular disease
T2D: type 2 diabetes
CRC: colorectal cancer
MI: myocardial infarction
mMED: modified Mediterranean diet score
HNFI: Healthy Nordic Food Index
GHG: greenhouse gas emission
EH: environmental hourglass.
